# Fecal Microbiota Transplantation Modulates Renal Phenotype in the Humanized Mouse Model of IgA Nephropathy

**DOI:** 10.3389/fimmu.2021.694787

**Published:** 2021-10-12

**Authors:** Gabriella Lauriero, Lilia Abbad, Mirco Vacca, Giuseppe Celano, Jonathan M. Chemouny, Maria Calasso, Laureline Berthelot, Loreto Gesualdo, Maria De Angelis, Renato C. Monteiro

**Affiliations:** ^1^ Center for Research on Inflammation, Inflamex Laboratory of Excellence, Paris University, Paris, France; ^2^ INSERM U1149, Paris, France; ^3^ CNRS ERL8252, Paris, France; ^4^ Nephrology, Dialysis and Transplantation Unit, Department of Emergency and Organ Transplantation, University of Bari Aldo Moro, Bari, Italy; ^5^ Department of Soil, Plant and Food Sciences, University of Bari Aldo Moro, Bari, Italy

**Keywords:** fecal microbiota transplantation, gut microbiota, BAFF, IgA nephropathy, mouse model

## Abstract

Immunoglobulin A nephropathy (IgAN) is the most common primary glomerulonephritis. Several observations suggest that gut microbiota could be implicated in IgAN pathophysiology. Aiming at exploring whether microbiota modulation is able to influence disease outcome, we performed fecal microbiota transplantation (FMT) from healthy controls (HC-sbjs), non-progressor (NP-pts) and progressor (P-pts) IgAN patients to antibiotic-treated humanized IgAN mice (α1KI-CD89Tg), by oral gavage. FMT was able to modulate renal phenotype and inflammation. On one hand, the microbiota from P-pts was able to induce an increase of serum BAFF and galactose deficient-IgA1 levels and a decrease of CD89 cell surface expression on blood CD11b^+^ cells which was associated with soluble CD89 and IgA1 mesangial deposits. On the other hand, the microbiota from HC-sbjs was able to induce a reduction of albuminuria immediately after gavage, an increased cell surface expression of CD89 on blood CD11b^+^ cells and a decreased expression of KC chemokine in kidney. Higher serum BAFF levels were found in mice subjected to FMT from IgAN patients. The main bacterial phyla composition and volatile organic compounds profile significantly differed in mouse gut microbiota. Microbiota modulation by FMT influences IgAN phenotype opening new avenues for therapeutic approaches in IgAN.

## Introduction

IgA nephropathy (IgAN) is the most common primary glomerulonephritis throughout most developed countries of the world (1) and it shows a progression to end-stage renal disease (ESRD) in approximately 15 to 25 percent of cases at 10 years (2). Hallmark of the disease is the presence of mesangial deposition of polymeric IgA1 (3).

In IgAN patients elevated levels of circulating galactose-deficient IgA1 (Gd-IgA1) elicit an autoimmune response and lead to the formation of immune complexes (3–5). Moreover, increased IgA1 complexes lead to the shedding of IgA Fcα receptor (CD89) expressed by monocytes and the integration of the soluble part of the receptor (sCD89) into the complexes (6). Decreased serum IgA1-sCD89 complexes have been shown to be associated with disease progression or recurrence after transplantation (7–9). IgA1 complexes deposit in the glomerular mesangium, where they activate mesangial cells through IgA receptor, transferrin receptor 1 (TfR1) (10–12).

In spite of a large number of investigations, pathogenesis of IgAN is only partially defined and the triggering event is still to be identified. A particular interest has been recently raised for the intestinal ecosystem. An extraordinary high number of microbes are harvested in the gut, collectively called microbiota. Gut microbiota controls the recruitment, differentiation, and function of innate and adaptive immune cells in mucosa-associated lymphoid tissue (MALT), acting both locally and systemically (13). The intestinal immune system is a unique environment that assures protection against pathogens through the production of IgA, maintaining tolerance to dietary proteins and commensal microbiota (14). IgA production can be induced by T-cell dependent or independent pathways, which occur in MALT (15). T-cell independent IgA class switching of B cells is induced by various cytokines but mainly by BAFF (B-cell activation factor) and APRIL (a proliferation-inducing ligand) (15, 16). Moreover, BAFF was shown to promote proliferation of human mesangial cells (17).

Several observations suggest that gut microbiota could be implicated in IgAN pathophysiology through a mucosal-kidney axis.

Firstly, from an experimental point of view, microbiota implication in IgAN is suggested by the observation that BAFF transgenic mice show high levels of aberrantly glycosylated serum polymeric IgA leading to mesangial deposition, the presence of commensal flora and commensal bacteria-reactive IgA antibodies being essential for the development of IgA deposits (18). A subgroup of patients with IgAN has elevated serum levels of APRIL (18). Under germ-free conditions, in mice expressing the human α1 heavy chain instead of the murine µ chain, glomerular IgA1 deposits are reduced, along with a significant reduction in serum IgA1 (19). In both these models, mice have been deprived of stimulation by commensal flora from birth, preventing the normal maturation of immune system (20).

We have previously characterized a humanized mouse model of IgA nephropathy, the α1KI-CD89Tg mouse (21), that overexpresses both human CD89 IgA receptor on myeloid cells and human IgA1 and develops circulating hIgA1-sCD89 complexes with mesangial IgA1 deposits associated with clinical features of renal injury including hematuria and proteinuria. Recently, we showed that microbiota depletion by antibiotic treatment of α1KI-CD89Tg mice prevented IgA1 mesangial deposition, glomerular inflammation and the development of proteinuria without affecting serum IgA levels (22), suggesting that nephritogenic IgA is modulated by the microbiota and originates from the gut (22, 23).

These data are supported by results of several genome-wide association studies (GWAS) performed on IgAN patients revealing new loci associated with risk of inflammatory bowel disease or maintenance of intestinal barrier and MALT response to pathogens (24). In addition, treatment with corticosteroids targeting the gut mucosa protects renal function in patients with IgAN (25).

We previously showed that some traits of gut microbiota significantly varied between healthy control subjects, non-progressor and progressor IgAN patients and that urinary and fecal metabolome consistently differed between groups (26).

With the aim of studying whether microbiota transplantation is able to influence disease expression, we transplanted intestinal microbiota from healthy control subjects (HC-sbjs), non-progressor and progressor patients (NP-pts and P-pts, respectively) into antibiotic-treated humanized IgAN mouse model, the α1KI-CD89Tg mouse. To the best of our knowledge this is the first study that applies fecal transplantation to a model of IgA nephropathy. Microbiota modulation by FMT from patients with severe disease influenced IgAN phenotype.

## Materials and Methods

### Human Fecal Microbiota Analysis

Fecal samples from IgAN patients and HC-sbjs were collected at the out-patient setting of the “Division of Nephrology, Dialysis and Transplant, Policlinico Hospital” of Bari, Italy. Exclusion criteria were described by De Angelis et al. (26).

At the time of enrollment, we evaluated serum creatinine, 24h-proteinuria and Glomerular Filtration Rate (GFR), estimated by The Modification of Diet in Renal Disease Study (MDRD) equation, and we repeated the evaluation after six months of follow-up.

As described by Rocchetti et al. (27), at the end of follow-up, IgAN patients were classified NP-pts and P-pts according to 24h-proteinuria and renal function. In detail, IgAN patients were classified as NP-pts if they showed a stable renal function and at least 50% decrease in daily proteinuria compared with previous evaluation. On the contrary, if they showed a decline of renal function, 24h-proteinuria ≥1 gr and/or at least 50% increase in daily proteinuria we classified them as P-pts. Main clinical features of IgAN patients and HC-sbjs included in the study are summarized in **Supplementary Table 1**. No significant difference in age and sex distribution was observed among subjects.

Each volunteer provided three fecal samples for a time span of three weeks. Bacterial tag-encoded FLX-titanium amplicon pyrosequencing of the 16S rRNA was carried out, as described by De Angelis et al. (26).

### ELISA Assay for Soluble Human BAFF Levels in Serum

Human BAFF serum levels from IgAN patients and HC-sbjs were quantified by using R&D Systems ELISA Kit, according to the manufacturer’s instructions.

### Study Design and *In Vivo* Experiments

The double transgenic α1KI-CD89Tg mice were raised and maintained in a specific pathogen-free mouse facility at the Centre for Research on Inflammation, Paris, France with free access to autoclaved food. All experiments were performed in accordance with French Council of animal care guidelines and National Ethics Guidelines and with approval of the Local Ethics Committee of Paris-Nord animal care committee (Animal use protocol number C2EA-121).

Mice remained with their mother for suckling until four-week-old. Then, male mice were randomly assigned to receive or not a broad-spectrum antibiotic mix. Five male mice did not receive antibiotic treatment and constituted the control group (**Figure 2A**). 

Twenty-nine four-week-old male mice were subjected to a four-week broad-spectrum antibiotic treatment in order to remove the commensal intestinal microbiota. Mice were treated by adding ampicillin (1 g/L; Panpharma), vancomycin (500 mg/L; Mylan), geneticin (1 g/L; Euromedex), metronidazole (1 g/L; Braun) and sucrose (75 g/L as sweetener) to the drinking water *ad libitum* for four weeks according to a standard protocol (28). All twenty-nine mice survived the antibiotic treatment.

The antibiotic mixture was replaced by sterile drinking water and starting four days after discontinuation of antibiotics (29), mice were fed by gavage with 0.2 ml of water or pooled microbial cell suspensions (bacterial load of 10^8^ CFU/ml) from fecal samples of HC-sbjs, NP-pts and P-pts for five consecutive days.

Four weeks after microbiota reconstitution, after anesthesia, blood was collected by cardiac puncture and thirteen-week-old mice were sacrificed by cervical dislocation.

Urine and feces were collected all over the experiment and stored at -20°C and -80°C, respectively.

At the day of sacrifice, perfused kidneys were collected. One kidney was embedded in O.C.T. freezing medium (CML, Nemours, France), snap frozen in liquid nitrogen. The other kidney was cut into two halves. The first half was fixed in 4% formalin (Sigma-Aldrich) and embedded in paraffin. The remaining renal tissue was frozen for mRNA extraction. Small and large intestines were frozen at -80°C until further processing for microbiological analysis.

### Kidney Functional Parameters

Albumin and creatinine levels were measured in urines using the AU400 chemistry analyzer (Olympus). For hematuria, 10 µl of fresh urines were mounted on a Malassez hemocytometer, and red cells were blind counted.

### Enzyme-Linked Immunosorbent Assay

Serum IgA and BAFF levels were respectively quantified by using a human IgA ELISA quantitation set (Bethyl, E80-102), a mouse BAFF Quantikine ELISA (RD Systems, catalog number DY2106-05) according to the manufacturer’s instructions. Measurement of hIgA1-sCD89 complexes was determined with a sandwich enzyme-linked immunosorbent assay (30). A3 anti-human CD89 mAb (10 μg/ml) was used for coating as described (21). Sera were then added and revealed with anti-human IgA (1:2000 dilution) coupled with alkaline phosphatase (Southern Biotech, Birmingham, AL, USA). The optic density (OD) at 405 nm was measured after the addition of the AP substrate (SIGMAFAST p-nitrophenyl phosphate tablets; Sigma-Aldrich). IgA1 glycosylation was assessed by a lectin ELISA using Helix aspersa agglutinin (HAA). Briefly, plates were coated overnight at 4°C with 10 μg/ml anti-human IgA (Bethyl Laboratories, Montgomery, TX, USA). After 8 h of blocking, the serum samples were adjusted to 10 μg/ml of IgA and incubated in the plate overnight at 4°C. To remove sialic acids, neuraminidase (from V. Cholerae, Roche, Boulogne-Billancourt, France) was added and incubated for 3 h at 37°C, followed by the addition of biotinylated Helix aspersa agglutinin (Sigma-Aldrich, Saint-Quentin Fallavier, France; 1/100) and an additional 3 h incubation at 37°C. After 30 min incubation with streptavidin–alkaline phosphatase, the alkaline phosphatase substrate was added, and plates were read at 405 nm. The lectin reactivity was expressed as the ratio of sample optical density (OD) to positive control OD. The positive control was a purified IgA1 in which galactose and neuraminic acid had been enzymatically removed earlier.

### Histological and Immunostaining Procedures

Four µm paraffin-embedded kidney sections were stained with Masson’s trichrome for morphological analysis. For immunohistochemistry, 4 µm sections of frozen kidney were fixed in acetone, incubated for 1 h in 5% bovine serum albumin (Euromedex, Souffelweyersheim, France), followed by 1 h 30 min at room temperature with biotinylated mouse anti-human IgA (Catalogue number 555884) or anti-mouse CD11b (Catalogue number 553309) (both from BD Biosciences, Le Pont de Claix, France). Detection was performed with vectastain elite ABCkit (Vector, Burlingame, CA, USA). Slides were mounted with Immuno-mount (Thermo Fisher Scientific), read with an upright microscope, DM2000 (Leica, Wetzlar, Germany) at ×400 magnification using IM50 software (Leica) and scanned with AperioScanScope CS System (Leica Microsystems SAS, Nanterre France) and staining was quantified using TRIBVN CaloPix software (TRIBVN, Chatillon France). For CD89 kidney staining, 4 µm sections of frozen kidney were fixed in acetone, incubated for 1 h in 5% bovine serum albumin, followed by anti-CD89 mAb (clone A3, homemade) and developed by staining with anti-mouse Ig coupled with FITC. Slides were mounted with Immuno-mount, read with an upright fluorescence microscope (Leica, Wetzlar, Germany) at ×40 magnification using IM50 software (Leica).

### Flow Cytometry

Heparinized whole blood samples were collected by cardiac puncture. Red blood cells from blood were lysed with RBC lysing buffer (BD Pharm LyseTM). All cells were re-suspended in buffer (phosphate-buffered saline (PBS) containing 1% Bovine Serum Albumin and 0.1% NaN3 for flow cytometry analysis). Cells from blood samples of α1KI-CD89Tg mice were stained with anti-mouse CD11b-PE-Cy7 and mouse CD89-PE antibodies (BD Biosciences). Samples were analyzed by Canto II flow cytometer (BD Biosciences).

### Quantitative Real-Time PCR Analysis

Total RNA from mouse kidneys was extracted using RNABLE (Eurobio), and reverse transcription was carried out using 1 μg of total RNA and Moloney murine leukemia virus reverse transcriptase (Invitrogen). The quantitative transcription profile of TNF-α, TGF-β, MIP-2, MCP-1 and KC expression in kidneys was determined in triplicate with a TaqMan real-time PCR assay using a CFX96 PCR system (Bio-Rad). PCR data were reported as the relative increase in mRNA transcripts using the levels of β-actin mRNA as normalizer. Primers for TNF-α and TGF-β are listed in **Supplementary Methods**; all other primer sequences are available in Rosatto et al. (31).

### Bacterial Microbiome Estimated by 16S rRNA Sequencing

Collected luminal contents from small and large intestines of mice were added with PBS (w/w 1:5), homogenized with Interscience-BagMixer (210”), and filtered (< 250 µm). An aliquot of 200 mg of sample was used for RNA extraction with the total RNA purification kit (Norgen Biotek Corp., Ontario, Canada). The resulting total RNA (ca. 2.5 μg) was transcribed to cDNA using random hexamers and the Tetro cDNA synthesis kit (Bioline USA, Inc., Taunton, MA, USA), according to the manufacturer’s instructions. 16S rRNA sequencing was carried out at Genomix4life (spin-off of the University of Salerno, Italy) by using the Illumina MiSeq platform. The V3-V4 region of the 16S rRNA gene for analysis of diversity inside the domains of Bacteria was amplified (32). The sequences of primers are: 16S Amplicon PCR Forward Primer = 5’ TCGTCGGCAGCGTCAGATGTGTATAAGAGACAGCCTACGGGNGGCWGCAG. 16S Amplicon PCR ReversePrimer=5’GTCTCGTGGGCTCGGAGATGTGTATAAGAGACAGGACTACHVGGGTATCTAATCC. Forward overhang: 5’ TCGTCGGCAGCGTCAGATGTGTATAAGAGACAG-[locus specific sequence]. Reverse overhang: 5’ GTCTCGTGGGCTCGGAGATGTGTATAAGAGACAG-[locus specific sequence]. PCR and sequencing analyses were carried out according to the protocol of Genomix4life. Quality control (QC) and taxonomic assignments were undertaken according to the QIIME and the Ribosomal Database Project Bayesian classifier in combination with a set of custom designed informatics pipelines implemented by Genomix4life for analyses of microbial communities. Taxonomic attribution was carried out using the BLAST search in the NCBI 16S rRNA sequences database (33). The percentage of each bacterial OTU was analyzed individually for each sample (34). Alpha-diversity indexes (including: number of OTUs, Chao1 species richness, and Shannon index) were calculated using QIIME (35, 36). The raw sequences were deposited in NCBI Sequence Read Archive (BioProject accession number: PRJNA732260; https://www.ncbi.nlm.nih.gov/sra/PRJNA732260).

### Analysis of Volatile Organic Compounds

Fecal samples from thirteen-week-old mice were collected to evaluate the volatile organic compounds (VOCs) profile. To obtain the best extraction efficiency, the micro-extraction procedure was performed as described in Carroccio et al. (37), with slight modifications. One gram of sample with addiction of 10 μl of 4-methyl-2-pentanol (final concentration 9.9 μg/g) was placed into 20 mL glass vials and sealed with polytetrafluoroethylene (PTFE)-coated silicone rubber septa (20-mm diameter; Supelco, Bellefonte, PA, USA). The samples were then equilibrated for 30 min at 60°C. At the end of sample equilibration, a conditioned 50/30 μm DVB/CAR/PDMS fibre (Supelco) was exposed to headspace for 50 min to extract volatile compounds by CombiPAL system injector autosampler (CTC Analytics, Zwingen, Switzerland). Volatile organic compounds (VOCs) were thermally desorbed by immediately transferring the fibre into the heated injection port (220°C) of a Clarus 680 (Perkin Elmer, Beaconsfield UK) gas chromatography equipped with an Rtx-WAX column (30 m × 0.25 mm i.d., 0.25 μm film thickness) (Restek) and coupled to a Clarus SQ8MS (Perkin Elmer) with source and transfer line temperatures kept at 250 and 210°C, respectively. The injection was carried out in splitless mode for two minutes, and helium was used as the carrier gas at flow rate of 1 mL/min. The oven temperature was initially set at 35°C for 8 min, then increased to 60°C at 4°C/min, to 160°C at 6°C/min, and finally to 200°C at 20°C/min and held for 15 min. Electron ionization masses were recorded at 70 eV in the mass-to-charge ratio interval, which was m/z 34 to 350. The GC-MS generated a chromatogram with peaks representing individual compounds. Each chromatogram was analyzed for peak identification using the National Institute of Standard and Technology 2008 (NIST) library. A peak area threshold of >1 000 000 and 90% or greater probability of matches was used for VOCs identification, followed by manual visual inspection of the fragment patterns when required. In order to quantify the identified compounds, the internal standard area was used by interpolation with the area of each compound. Final concentrations were expressed as μg/g of 2-methyl-4-pentanol.

### Statistics

Kruskal-Wallis test with Dunn’s multiple comparisons test or Mann-Whitney U test were performed for comparisons between groups. Correlations were analyzed with Spearman’s test. Statistical analyses were performed with GraphPad Prism 6.0 (GraphPad Software, Inc., San Diego, CA).

For the microbiological analysis, differences between sampled groups were analyzed using the two-tailed Student’s t-test for independent samples, with a significance level of p-value (P) ≤ 0.05. For metabolomic analysis, one-way ANOVA analysis of variance with a post-hoc Tukey test was performed for comparisons between groups. Permut-MatrixEN software was used to identify clusters among the sampled groups and taxa (38).

### Study Approval

The human study was approved by the “Policlinico Hospital” of Bari (Italy) Ethics Committee (protocol number 606) with written informed consent obtained from each participant. The study was conducted according to the criteria set by the declaration of Helsinki.

Animal experiments were performed in accordance with French Council of animal care guidelines and National Ethics Guidelines and with approval of the Local Ethics Committee of Paris-Nord animal care committee (Animal use protocol number C2EA-121).

## Results

### Fecal Microbiota Composition and Serum BAFF Levels in IgAN Patients and Healthy Controls

Transgenic humanized α1KI-CD89Tg mice spontaneously develop mesangial IgA1 deposition, along with proteinuria, mimicking human IgA nephropathy (21). We showed that microbiota depletion by antibiotic treatment prevent IgA nephropathy development in these mice (22), confirming that nephritogenic IgA is modulated by a microbiota gut-kidney axis (23). To explore whether the different intestinal microbiota composition in HC-sbjs, NP-pts and P-pts could alter renal disease progression, we decided to perform fecal microbiota transplantation in humanized IgAN α1KI-CD89Tg mice.

Firstly, we collected stools and sera from IgAN patients and healthy control subjects. 16S rRNA gene sequencing analysis was performed to confirm that, at genus and phylum level, the differences in the composition of metabolically active bacteria between groups was similar to what previously reported (26) (**Supplementary Figure 1**). In particular, metabolically active Firmicutes increased in NP-pts (65.15%) and P-pts (63.18%) compared to HC-sbjs (58.94%; data not shown). An opposite trend was found for Bacteroidetes, which showed the highest value in HC-sbjs (data not shown).

**Figure 1:**
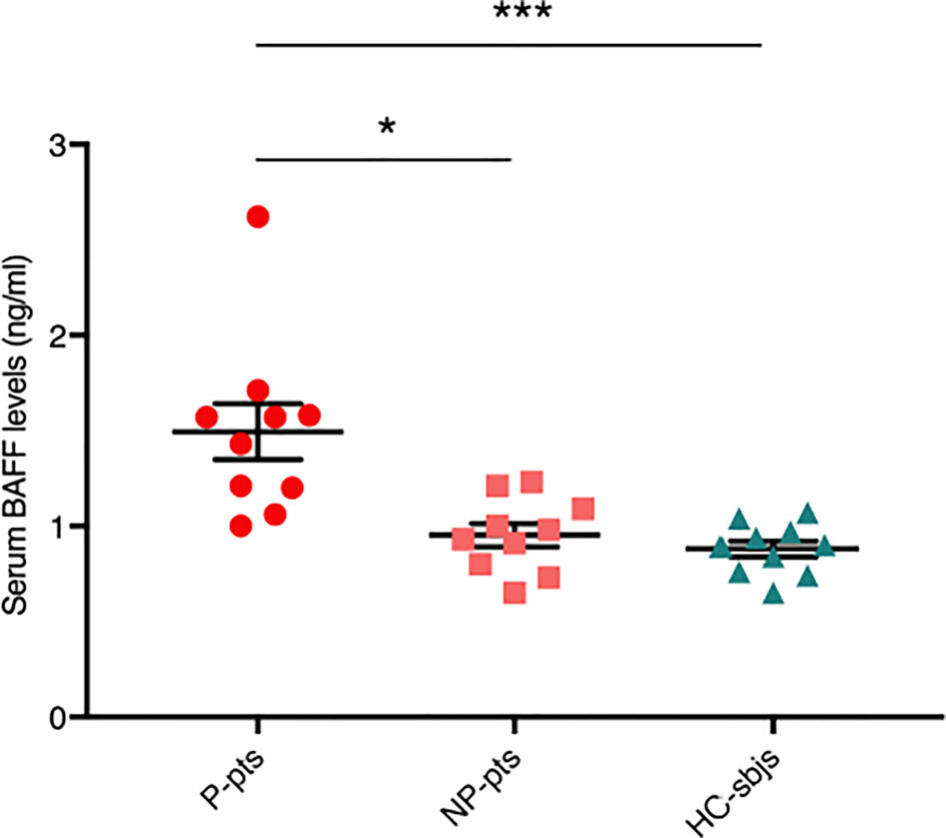
Serum BAFF levels in fecal microbiota donors. Serum BAFF levels were evaluated in healthy control subjects (HC-sbjs) and in progressor and non-progressor IgAN patients (P-pts and NP-pts, respectively) by ELISA. BAFF levels were significantly higher in P-pts than both HC-sbjs (***P ≤ 0.001) and NP-pts (*P ≤ 0.05; Kruskal-Wallis test with Dunn’s multiple comparisons test).

We measured serum BAFF levels in fecal microbiota donors and found that they were significantly higher in P-pts (mean ± standard error of mean (SEM), 1.5 ± 0.15 ng/ml) compared to HC-sbjs (0.88 ± 0.04 ng/ml; P ≤ 0.001) and NP-pts (0.95 ± 0.06 ng/ml; P ≤ 0.05) (**Figure 1**). BAFF is a cytokine involved in T-cell independent IgA class switching of B cells (15, 16) and it was shown to promote proliferation of human mesangial cells (17).

### Fecal Microbiota Transplantation Modulates Renal Phenotype in the Humanized Mouse Model of IgAN

**Figure 2:**
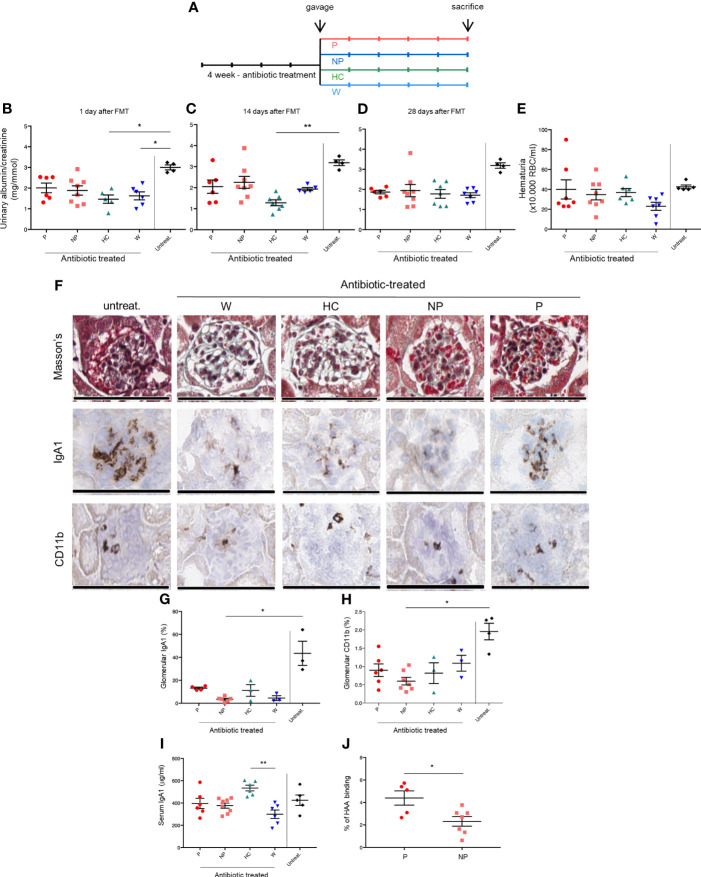
FMT modulates renal phenotype in the humanized mouse model of IgAN. **(A)** Study design. Twenty-nine 4-week-old male of α1KI-CD89Tg mice were subjected to 4 weeks of broad-spectrum antibiotic treatment in order to remove the commensal intestinal microbiota. The antibiotic mixture was replaced by sterile drinking water and starting four days after discontinuation of antibiotics, mice were fed by gavage with water (W mice) or pooled microbial cell suspensions from fecal samples of healthy control subjects, non-progressor and progressor IgAN patients (HC, NP and P mice, respectively) for five consecutive days. Four weeks after microbiota reconstitution, thirteen-week-old mice were sacrificed. Five male mice did not receive antibiotic treatment and they constituted the control group (untreat.). **(B)** Albumin to creatinine ratio measured in the urines of mice at the end of gavage. **(C)** Albumin to creatinine ratio measured in the urines of mice at 2 weeks after gavage. **(D)** Albumin to creatinine ratio measured in the urines of 13-week-old mice (at 4 weeks after gavage). **(E)** Hematuria (×10.000 red blood cells/ml) in 13-week-old mice. **(F)** From the top to the bottom: Masson’s trichrome staining of paraffin-embedded kidney sections, immunostaining for human IgA1 and CD11b^+^ infiltrates in frozen kidney sections of mice. Bars: 100 μm. Quantification of glomerular IgA1 deposits **(G)** and CD11b+ infiltrates **(H)**. **(I)** Serum human IgA1 levels in 13-week-old mice measured by ELISA. **(J)** Percentages of HAA binding on circulating IgA corresponding to galactose deficient-IgA1 levels in 13-week-old P and NP mice (Mann-Whitney U test). HAA: helix aspersa agglutinin. Bars and error bars represent the mean ± SEM. *P ≤ 0.05; **P ≤ 0.01 (Kruskal-Wallis test with Dunn’s multiple comparisons test).

To evaluate whether transplanting intestinal microbiota leads to a different disease progression, we exposed α1KI-CD89Tg mice to a-four-week-course of broad-spectrum antibiotic treatment in order to remove the commensal intestinal microbiota before fecal microbiota transplantation (FMT) by oral gavage. Four weeks after microbiota reconstitution, thirteen-week-old mice were sacrificed (**Figure 2A**).

**Figure 3:**
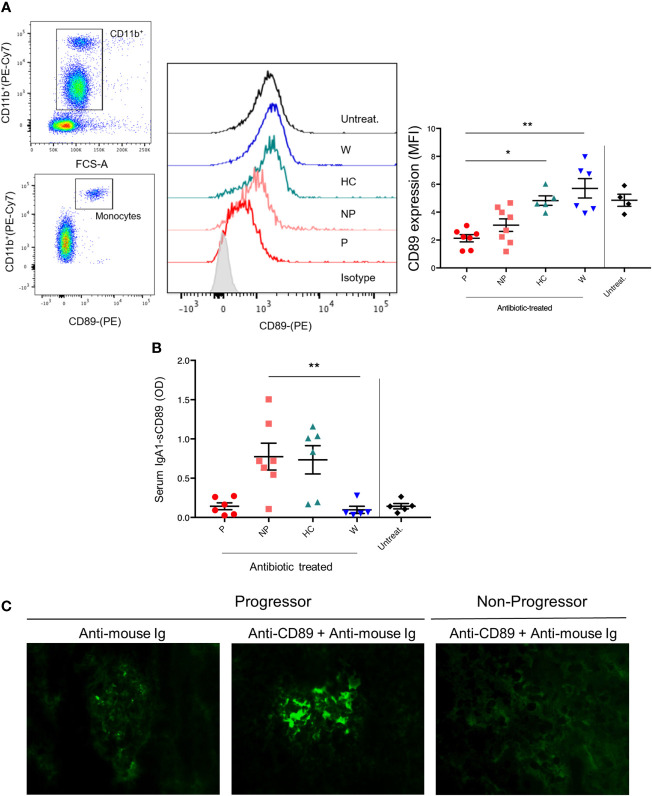
**(A)** Getting strategy and representative flow cytometry profiles with quantification of CD89 expression on blood CD11b^+^ cells were shown. MFI, mean fluorescence intensity. **(B)** Serum IgA1–sCD89 complexes were detected by ELISA. O.D., optical density. Bars and error bars represent the mean ± SEM. *P ≤ 0.05; **P ≤ 0.01(Kruskal-Wallis test with Dunn’s multiple comparisons test). **(C)** Immunostaining of kidney cryostat sections from P and NP mice using anti-CD89 mouse monoclonal antibody (clone A3) plus a secondary anti-mouse antibody coupled to FITC. Negative control was performed with secondary Ab alone.

**Figure 4:**
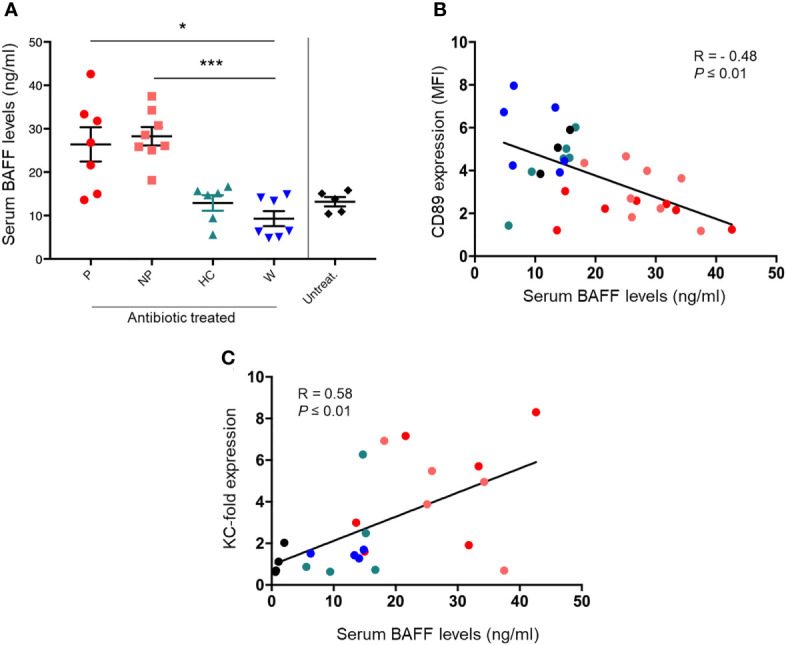
FMT was able to modulate serum BAFF levels in the humanized mouse model of IgAN. **(A)** Serum BAFF levels in mice fed by gavage with water (W) or pooled microbial cell suspensions from fecal samples of HC-sbjs, P-pts and NP-pts, were detected by ELISA. Bars and error bars represent the mean ± SEM. *P ≤ 0.05; ***P ≤ 0.001 (Kruskal-Wallis test with Dunn’s multiple comparisons test). **(B)** Correlation analysis between serum BAFF levels and cell surface expression of CD89 on blood CD11b^+^ cells **(C)** Correlation analysis between serum BAFF levels and relative KC mRNA expression in kidney.

Urinary albumin to creatinine ratio (ACR) was measured at the end of the gavage (**Figure 2B**) and 2 to 4 weeks later (**Figure 2C, D**). As expected (22), mice treated with antibiotics only (gavage with water; W mice) showed a decreased ACR at the end of gavage, confirming that host microbiota depletion protects from IgAN progression in α1KI-CD89Tg mice. A trend in ACR reduction was maintained for all groups compared to untreated mice, but in particular the group subjected to FMT from HC-sbjs (HC mice) showed a significant reduction in ACR, both at the end of gavage (**Figure 2B**) and at two weeks after gavage (**Figure 2C**), suggesting that a healthy microbiota may delay IgAN nephropathy progression. Hematuria did not differ between groups, as evaluated at the end of the experiment (**Figure 2E**).

Histologically, glomeruli in mice fed with water or fecal microbial cell suspensions from IgAN patients, but not from healthy controls, showed mesangial expansion and appeared to be richer in red blood cells (**Figure 2F**).

In IgAN patients, CD89, the myeloid Fc receptor for IgA, sheds from myeloid cell surfaces following interaction with aberrant IgA resulting in the formation of circulating complexes containing IgA1 and soluble CD89 (sCD89) that deposit in kidney mesangium (6, 21).

Severity of renal dysfunction in IgAN patients was reported to be correlated with the disappearance of IgA1–sCD89 complexes from the circulation, suggesting that this could be caused by their increased deposition in kidney (8, 39).

Therefore, we analyzed serum hIgA1 and Gd-hIgA1 levels (evaluated by HAA binding) as well as mesangial hIgA1 deposits and infiltrating CD11b^+^ cells in the glomeruli. While myeloid infiltration and hIgA1 deposition was globally reduced in antibiotic-treated mice, we observed a trend towards lower levels in mice subjected to FMT from NP-pts (NP mice) compared with mice subjected to FMT from P-pts (P mice) (**Figures 2F–H**). Although serum IgA1 concentrations were not significantly different between P and NP mice (**Figure 2I**), circulating Gd-hIgA1 were significantly higher in P compared with NP mice (P ≤ 0.05; **Figure 2J**).

In order to explore the role of soluble CD89, we first analyzed CD89 surface expression in circulating CD11b^+^ cells. As shown in **Figures 3A, B**, mice treated with FMT from P-pts (P mice) showed the lowest levels of CD89 cell surface expression on blood CD11b^+^ cells compared with both W and HC mice (P ≤ 0.01 and P ≤ 0.05, respectively) and a trend for decreased serum hIgA1-sCD89 complexes, suggesting that FMT from P-pts enhanced CD89 shedding. This could promote hIgA1-sCD89 complex formation leading to deposits in the kidney, as suggested by an observed trend of increased hIgA1 glomerular deposits in P-mice as compared to NP-mice. Inversely, HC and W mice showed the highest levels of CD89 expression in CD11b^+^ cells, suggesting a reduced CD89 shedding process. However, levels of serum hIgA1–sCD89 complexes were significantly increased in NP mice when compared with W mice (P ≤ 0.01; **Figure 3B**). To address whether hIgA1-sCD89 complexes were deposited in the kidneys, we stained sections with an anti-CD89 mouse monoclonal antibody (clone A3). As shown in **Figure 3C**, sCD89 deposits were found in the mesangial area of glomeruli in P mice but not in NP ones. Levels of sCD89 deposits were similar to untreated animals (**Supplementary Figure 2**).

In order to examine local inflammation in kidney tissues, we assessed chemokines and cytokine mRNA levels in kidney extracts by quantitative PCR. KC is a secreted factor that acts as a neutrophil chemoattractant (40, 41) and was found to stimulate proliferation in renal epithelial cells (42). It is also upregulated during renal injury (43) where it has been proposed to play a role in the process of neutrophil infiltration and subsequent inflammation. KC receptor is expressed both on renal parenchymal cells as well as neutrophils (44, 45). A study showed that colon-kidney cross talk in DSS-induced acute colitis was mediated by KC receptor (46). Genetic deletion of KC receptor in mice suppressed inflammation and acute kidney injury, whereas administration of recombinant KC exacerbated acute kidney injury (46). KC expression resulted to be decreased in kidneys of HC mice compared with both P and NP mice (P ≤ 0.01; **Supplementary Figure 3**). This result suggests a decreased recruitment of inflammatory cells in kidneys of HC mice, in contrast to those of mice fed with fecal microbial cell suspensions from IgAN patients. No difference was found in levels of TNFα, MCP-1, TGFβ, MIP-2 (data not shown).

### Fecal Microbiota Transplantation Is Able to Modulate Serum BAFF Levels in the Humanized Mouse Model of IgAN

BAFF serum levels were significantly increased in both P and NP mice, compared with W mice (P ≤ 0.05 and P ≤ 0.001, respectively; **Figure 4A**). Moreover, BAFF levels correlated negatively with CD89 surface staining in blood CD11b^+^ cells (Spearman’s r = -0.48, P ≤ 0.01; **Figure 4B**). A positive correlation was found between serum BAFF levels and KC expression in kidneys (Spearman’s r = 0.58, P ≤ 0.01; **Figure 4C**).

### Fecal Microbiota Transplantation Is Able to Modulate Mouse Gut Microbiota Composition

Total bacteria from small intestine (SI) and large intestine (LI) of mice were analyzed by 16S rRNA gene sequencing. The bacterial community was analyzed at species level (OTUs), richness estimator (Chao1) and diversity index (Shannon) (**Supplementary Table 2**).

**Figure 5:**
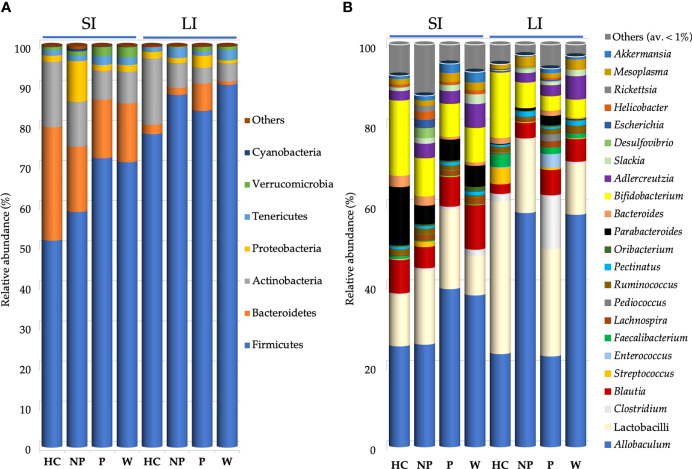
Bacterial composition found in mouse gut microbiota after FMT. Mean of relative abundance (%) of bacteria at phylum **(A)** and at genus **(B)** level found in small intestine (SI) and large intestine (LI) of 13-week-old α1KICD89Tg mice fed with water (W mice) or pooled microbial cell suspensions from fecal samples of healthy controls (HC mice), progressor and non-progressor IgAN patients (P and NP mice, respectively). Phyla with relative abundance < 0.1% among all mice and genera with an average relative abundance < 1% were grouped together in Others.

Firmicutes, Bacteroidetes, Proteobacteria, Actinobacteria, Tenericutes, and Verrucomicrobia phyla represented more than 99% of 16S rRNA gene sequences (**Figure 5A**).

Bacteroidetes were decreased in the LI of W mice (0.81%) compared with P mice (7.79%; P ≤ 0.05; **Figure 5A**). However, the ratio Firmicutes/Bacteroidetes did not differ between mice groups.

Proteobacteria were increased in the LI of P mice (3.3%), compared with W (0.74%; P ≤ 0.001) and NP mice (1.22%; P ≤ 0.05; **Figure 5A**).

At genus level (**Figure 5B**), within *Lactobacillaceae*, lactobacilli were decreased in the SI of W mice (9.82%), particularly if compared with P mice (21.81%; P ≤ 0.05; **Supplementary Table 3**).

Within Bacteroidetes, *Bacteroides* spp. showed the highest relative abundance in SI of HC mice (2.78%) compared with both P mice (0.73%; P ≤ 0.05) and W mice (0.85%; P ≤ 0.05; **Supplementary Table 4**).

### Fecal Microbiota Transplantation Is Able to Modulate Volatile Organic Compounds Profile

**Figure 6:**
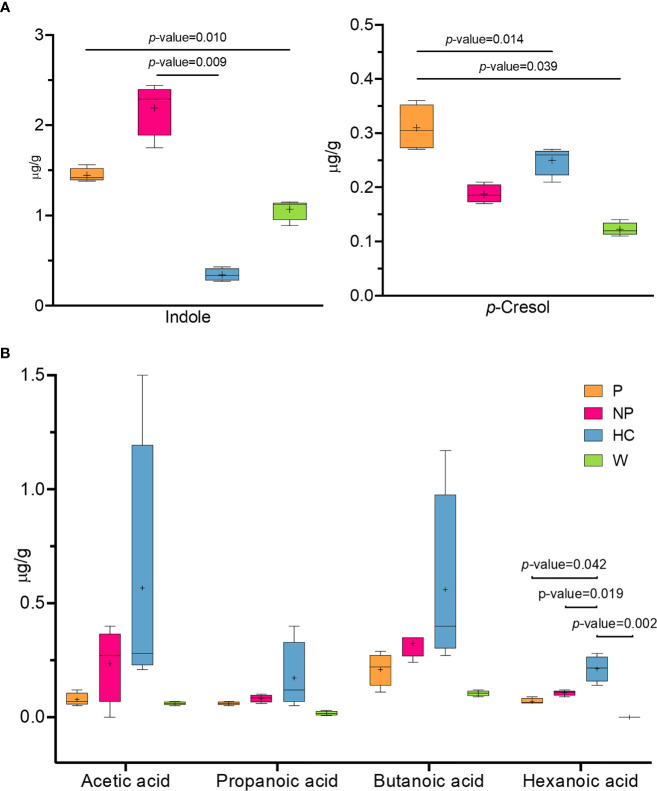
Fecal microbiota transplantation is able to modulate volatile organic compounds profile. Concentrantion of volatile organic compounds profile (VOCs) (μg/g) in fecal samples from 13-week-old α1KICD89Tg mice fed with water (W mice) or pooled microbial cell suspensions from fecal samples of healthy controls (HC mice), progressor and non-progressor IgAN patients (P and NP mice, respectively). **(A)** Comparison of indole and *p*-cresol levels and **(B)** SCFAs concentration.

A comparison of metabolomic patterns in fecal samples of mice after FMT was performed based on qualitative and quantitative differences in VOCs using HS-SPME GC–MS methodology. Fifty-nine volatile compounds were identified and grouped according to chemical classes, i.e., alcohols (14), aldehydes (11), organic acids (7), ketones (6), phenols (5), indoles (2), esters (3), terpenoids (3), hydrocarbons (3) and others (5) (data not shown). The content of metabolites largely varied among the groups; indeed, some significant differences were evaluated within alcohols, aldehydes, phenols, organic acids, ketones and indoles (**Supplementary Table 5**). Indole and *p*-cresol were significantly higher (P ≤ 0.05) in NP and P mice compared to HC mice (**Figure 6A**). Short chain fatty acids (SCFAs) were also evaluated in fecal samples of mice after FMT. Although no significant difference was observed in SCFAs content, a higher average amount of acetic acid, propanoic acid and butanoic acid was assessed in HC mice compared to other groups. On note, the level of hexanoic acid in P, NP and W mice were significantly reduced (P ≤ 0.05) compared to HC mice (**Figure 6B**).

### Microbiome Correlated With Some Phenotypic Features of Mice Subjected to Fecal Microbiota Transplantation

**Figure 7:**
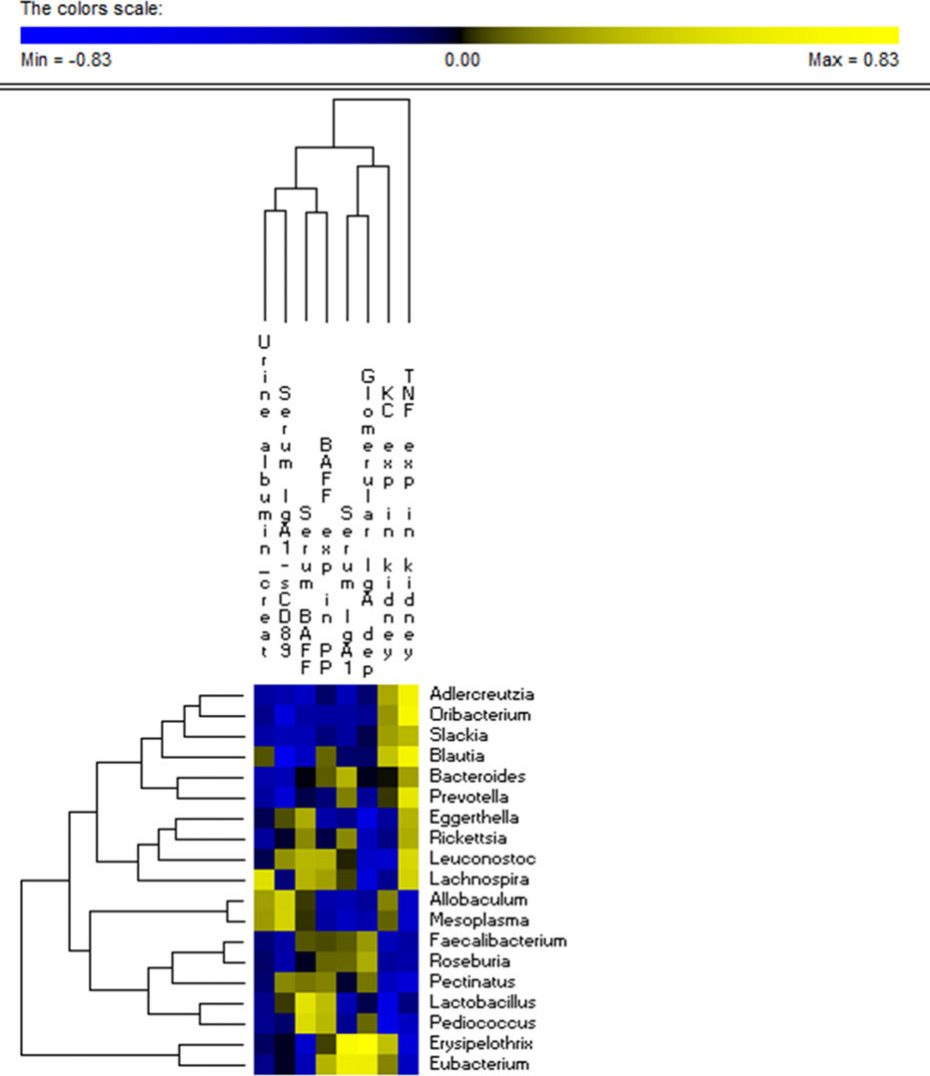
Microbiota correlation with some phenotypic features of mice subjected to FMT. Significant correlations between the relative abundance of bacterial genera found within all the four group of the analyzed 13-week-old α1KICD89Tg mice and the relative phenotypic features, in detail: urine albumin to creatinine ratio (urine albumin/creat), serum IgA1-sCD89, serum BAFF levels, KC chemokine expression in kidney (KC exp in kidney), serum IgA1, glomerular IgA1 deposits (glomerular IgA dep) and TNFα expression in kidney (TNF exp in kidney). The colors of the scale bar denote the degree of the correlation, from +0.83 (yellow) to −0.83 (blue). Only significant correlations (FDR ≤ 0.05) are shown.

Correlation analysis showed that *Erysipelothrix* and *Eubacterium* genera were positively correlated to both serum IgA1 levels (r = 0.71 and r = 0.59, respectively) and glomerular IgA1 deposits (r = 0.83 and r = 0.57, respectively; **Figure 7**). Both lactobacilli and pediococci showed a negative correlation with KC expression in kidney (r = -0.5 and -0.52, respectively; **Figure 7**). A direct correlation with TNFα expression in kidney was shown by *Adlercreutzia* (r = 0.63), *Prevotella* (r = 0.51), *Oribacterium* (r = 0.67) and *Blautia* (r = 0.65; **Figure 7**). The genus *Blautia* was negatively correlated with serum IgA1-sCD89 levels (r = -0.59; **Figure 7**).

## Discussion

At intestinal level, there is a dynamic cross-talk between the microbiota and the host immune system (13, 14). Intestinal microbiota acts both locally and systemically coordinating the organization and maturation of lymphoid tissues, which in turn can modulate the composition and function of gut microbiota (13, 14). Moreover, microbiota affects mucosal IgA production and IgA responses to microbiota seem essential for gut homeostasis (47). As microbiota depletion by broad spectrum antibiotics prevents or reverses the IgAN phenotype in α1KI-CD89Tg mice keeping normal levels of serum IgA (22), here we investigated whether transplanting intestinal microbiota through FMT procedures from HC-sbjs, NP-pts, and P-pts into antibiotic-treated humanized IgAN mouse model (α1KI-CD89Tg) leads to a different disease outcome.

Based on the results that we obtained, FMT is able to modulate renal phenotype. To date this is the first published study that applies FMT to IgAN.

Modulation of IgAN phenotype in mice by FMT was linked to the severity of the disease as fecal microbiota from P-pts was able to induce an increase of serum BAFF and Gd-IgA1 levels, and a decrease of CD89 cell surface expression on blood CD11b^+^ cells which was associated with soluble CD89 and IgA1 mesangial deposits. These data suggest that Gd-IgA1 induce shedding of CD89 to form nephritogenic hIgA1-sCD89 complexes. Of note, P mice displayed sCD89 mesangial deposits and very low levels of circulating hIgA1-sCD89 complexes, reproducing what had been observed in patients with recurrent IgAN after kidney transplantation (7).

On the other hand, microbiota from HC-sbjs was able to induce low albuminuria following FMT, an increased cell surface expression of CD89 on CD11b^+^ cells in peripheral blood suggesting a decrease in CD89 shedding and a low expression of KC chemokine in kidney.

Moreover, the microbiota from NP-pts induced an intermediate phenotype with a decreased amount of inflammatory cell infiltration in glomeruli and IgA1-mesangial deposition and high levels of serum hIgA1–sCD89 complexes, but with an increased expression of KC chemokine in kidney, increased serum BAFF levels and increased BAFF expression in PP. Interestingly, NP mice displayed no sCD89 deposits in the mesagium. Mice first treated with antibiotics and then fed with water (W mice), and not subjected to FMT, also showed an intermediate phenotype characterized by reduced albuminuria just after gavage, an increased cell surface expression of CD89 on CD11b^+^ cells in peripheral blood with low levels of serum hIgA1–sCD89 complexes, suggesting a decrease in CD89 shedding, and decreased serum BAFF levels. Taken together, these data suggest that microbiota composition is essential to determine the nephritogenic phenotype.

Moreover, our results confirm the key value of BAFF. BAFF is a cytokine member of the tumor necrosis factor (TNF) family involved in T cell–independent IgA class switching of B cells in the lamina propria (15, 48). Increased serum BAFF levels were found in several autoimmune diseases (49–53) and associated with the presence of autoantibodies (54, 55). BAFF has become a target for therapies of autoimmune diseases (54, 56–58). Serum BAFF levels were found to be weakly correlated with serum IgA levels in patients with IgAN undergoing tonsillectomy, and monocytes from patients with IgAN were found to produce more BAFF in response to CpG-ODN (59). Overexpression of BAFF in mice resulted in increased IgA levels within the intestinal lamina propria and IgA mesangial deposition, along with high circulating levels of polymeric IgA that is aberrantly glycosylated (18, 60). Type I and type II interferon triggered by toll-like receptors (TLR) could induce overexpression of BAFF in dendritic cells, favoring B cell expansion and increasing IgA synthesis (60). TLR9 activation has been shown to strongly up-regulate expression of TACI, a BAFF receptor (61). The IgA production by tonsillar mononuclear cells from patients with IgAN was up-regulated by TLR9 activation, and the up-regulation was inhibited by treatment with anti-BAFF antibody (59). Mucosal challenge with a TLR activator enhanced IgAN-specific phenomena, such as glomerular IgA deposition and elevation of serum IgA and the IgA-IgG2 immune complex (62). Moreover, BAFF enhanced TLR7/9 expression on B cells and TLR-mediated production of autoantibodies (61). Accordingly, BAFF may be one of the key cytokines linking TLR stimulation and IgAN development.

Analyzing serum BAFF levels in IgAN patients and HC-sbjs that we enrolled as volunteers for collection of fecal microbiota, we found that serum BAFF levels were increased in P-pts, compared with HC-sbjs, according with previous publications (63, 64) and differently from other studies (18, 65). Very interestingly, we found that FMT was able to modulate serum BAFF levels; serum BAFF levels in mice subjected to FMT showed the same trend of human serum BAFF levels; higher serum BAFF levels were found in mice fed with fecal microbial cell suspensions from IgAN patients.

Microbiome analysis revealed that the composition of main bacterial phyla significantly differed in mouse gut microbiota.

P, NP and W mice exhibited a decreased relative abundance of Bacteroidetes and a strong colonization of Firmicutes compared to HC mice, reproducing results described in humans (26, 66, 67), even if the ratio Bacteroidetes/Firmicutes did not differ between mice groups. According to other studies, an increase of relative abundance of Firmicutes and a related reduction of Bacteroidetes were associated to intestinal inflammation (68–70).

Moreover, the dysbiosis associated to chronic kidney disease (CKD) includes an increased abundance of Proteobacteria (71), as was observed in the large intestine of P mice. An enrichment of Proteobacteria was associated to inflammation facilitating gut colonization by exogenous pathogens (72). Indeed, DNA from *Enterobacter*, *Escherichia* and *Proteus* species was found in the blood of patients with ESRD (73).

In this study, collected intestinal tracts of HC mice showed an increased relative abundance of Actinobacteria (although the increase did not reach significance), indeed it was reported as the gut microbiota of CKD patients was characterized by low levels of Actinobacteria (71).

The effect of FMT from HC-sbjs, NP-pts, and P-pts was assessed on the volatile organic compounds in fecal samples of α1KI-CD89Tg mice. HC mice were characterized by an overall higher amount of SCFAs, as one of the key players in the metabolic interaction between kidney and gut microbiota. Several microbial taxa in the gut possess ability to produce SCFAs. Among these, *Bacteroides*, *Ruminococcaceae*, *Clostridia*, *Prevotella*, *Oscillospira* and *Akkermansia muciniphila* are commonly associated with increased production of SCFAs (74–76). In our study, a higher amount of *Bacteroides* was assessed in HC mice, suggesting that FMT from HC-sbjs may enhance the abundance of SCFAs producers. SCFAs exert their effects on the “entero-renal axis” mainly through G protein-coupled receptors (GPR) and the inhibition of histone deacetylase (HDAC) (77). SCFAs were found to inhibit the proliferation of glomerular mesangial cells induced by lipopolysaccharides (LPS) and high glucose in Gram-negative bacteria *via* GPR, and then reversed the production of reactive oxygen species (ROS) and malondialdehyde (MDA) (77, 78). In a recent study, the levels of acetic acid, propionic acid, butyric acid and hexanoic acid were significantly reduced in IgAN patients compared with control group (79). In detail, Chai et al. showed that hexanoic acid levels were negatively correlated with 24h proteinuria and positively correlated with serum albumin level. Along with the highest amount of hexanoic acid in HC mice (P ≤ 0.05), this could suggest a beneficial role of this compound in IgAN patients. On the other hand, the observation of higher levels of precursors of uremic toxins (i.e. indoxyl sulfate and *p*-cresyl sulfate) in P and NP mice was in line with another study on FMT in mice with CKD (80). In this study a decreased *p*-cresyl sulfate accumulation and improved glucose tolerance were found in mice with CKD after FMT from HC mice.

The correlation analysis showed that *Erysipelothrix* and *Eubacterium* genera were positively correlated with serum IgA1 levels and with the amount of IgA1 glomerular deposition. Previously, it was showed that bacterial taxa such as *Prevotellaceae* and *Helicobacter* were highly coated with IgA (81). The approach of purification and subsequent identification of IgA-coated bacteria in healthy wild-type C57BL/6 mice confirmed that a subset of taxonomically distinct bacteria retained bound IgA with sufficient affinity to withstand the dilution and washing steps intrinsic to the protocol (82). The authors confirmed that IgA-bound microbes form a subset within a complex microbiota and this subset would contain immunologically relevant microbes associated with inflammatory processes. Some of these microbes would be classified as pathobionts because they did not cause harm in their donor consortium.

Both lactobacilli and pediococci showed a negative correlation with chemokine KC expression in kidney. The group of lactobacilli was shown to slow the progression of kidney disease by improving the intestinal environment (83). In line with this, it was previously reported that lactobacilli secreted compounds that have been found to downregulate the expression of virulence factors acting at the brush border (84–86).

A positive correlation with TNFα expression in kidney was shown by *Adlercreutzia* (*Egghertellaceae*), *Prevotella (Prevotellaceae), Oribacterium* and *Blautia (Lachnospiraceae)*. In a previous work (87) we discussed the controversial role of *Lachnospiraceae*, a bacterial family mainly associated to healthy states. However, it is not the first time that some members of *Lachnospiraceae* were enriched in inflammatory states. This bidirectional evidence remains stable also evaluating different studies on mice. In some cases, *Lachnospiraceae* were found increased in a subset of mice showing increased levels of TNFα (88), meanwhile in another trial the *Clostridium cluster* XIVa (i.e., *Lachnospiraceae*) showed a negative correlation with TNFα (89). In light of these findings, it is possible to speculate that the influence of microbes on inflammatory state and on inflammatory markers, including TNFα, needs to be evaluated analyzing the deeper taxonomic levels (i.e., species or strain level) rather than the highest ones (e.g., phylum or family level). Moreover, the obtained positive correlation requires an integrated point of view including the contribution of negatively correlated microbial patterns. Thus, although an increased relative abundance of some microbial patterns could affect the expression of specific cytokines, the overall contribution of microbiota did not reach significance according to the different FMTs.

Currently, there are no specific therapies for IgAN, so these results suggest promising new field of investigation for treatments targeting the microbiota and its modulation. Although specific antibiotics, probiotic and/or prebiotic preparations or diets [i.e. gluten-free diet (90, 91)] could be options to modulate intestinal microbiota and disease following-up the clinical progression, we are still far from having validated choices by controlled clinical trials. An ultimate strategy of microbiota modulation could be fecal transplantation, which has been shown to be efficient in treating *Clostridium difficile*-associated disease (92).

In conclusion, this first preclinical assay in a humanized mouse model expressing IgA1 and its CD89 receptor indicates that the microbiota transfer by FMT modulates the IgAN phenotype opening avenues for new therapeutic approaches for IgAN patients.

## Data Availability Statement

The datasets presented in this study can be found in online repositories. Repository: NCBI SRA. Accession to cite for SRA data: PRJNA732260. SRA records are accessible with the following link (https://www.ncbi.nlm.nih.gov/sra/PRJNA732260).

## Ethics Statement

The animal study was reviewed and approved by French Council of animal care guidelines and National Ethics Guidelines and with approval of the Local Ethics Committee of Paris-Nord animal care committee (Animal use protocol number C2EA-121). Written informed consent was obtained from the owners for the participation of their animals in this study.

## Author Contributions

RM and LG designed the study. GL, LA, MV, GC and MC carried out experiments. GL, LB and MDA analyzed the data. GL and MDA made the figures. GL and MDA drafted the paper. RM, LG, MDA, LB, LA and JC revised the paper. All authors contributed to the article and approved the submitted version

## Funding

This work was supported by INSERM, by FRM (Fondation pour la Recherche Médicale) grant: Equipe FRM DEQ20140329531 and by ANR-18-CE14-0030-01. GL was supported by the European Renal Association–European Dialysis and Transplant Association and the Italian Society of Nephrology.

## Conflict of Interest

The authors declare that the research was conducted in the absence of any commercial or financial relationships that could be construed as a potential conflict of interest.

## Publisher’s Note

All claims expressed in this article are solely those of the authors and do not necessarily represent those of their affiliated organizations, or those of the publisher, the editors and the reviewers. Any product that may be evaluated in this article, or claim that may be made by its manufacturer, is not guaranteed or endorsed by the publisher.
