# Supplementary material for: Fecal Microbiota Transplantation Modulates Renal Phenotype in the Humanized Mouse Model of IgA Nephropathy
Source: Front Immunol. 2021 Oct 12;12:694787. doi: 10.3389/fimmu.2021.694787 (PMC8546224; doi:10.3389/fimmu.2021.694787)
Supplement: Supplementary file 1 [file DataSheet_1.docx]

Supplementary Material

# Supplementary methods

## Quantitative real-time PCR analysis

TNFα and TGF-β primer sequences included: TNFα, forward, 5′-CAT CTT CTC AAA ATT CGA GTG ACA A-3′, reverse, 5′-TGG GAG TAG ACA AGG TAC AAC CC-3′; probe, FAM-5′-CAC GTC GTA GCA AAC CAC CAA GTG GA-3′-TAMRA; TGF-β, forward; 5′-TGA CGT CAC TGG AGT TGT ACG G-3′, reverse, 5′-GGT TCA TGT CAT GGA TGG TGC-3′, FAM-5′-TTC AGC GCT CAC TGC TCT TGT GAC AG-3′-TAMRA.

# Supplementary Figures and Tables

## Supplementary Figures

**Supplementary Figure 1.** Relative abundance (average, %) of microbial genera found in fecal samples of healthy control subjects (HC-sbjs), non-progressor (NP-pts), and progressor (P-pts) IgAN patients.


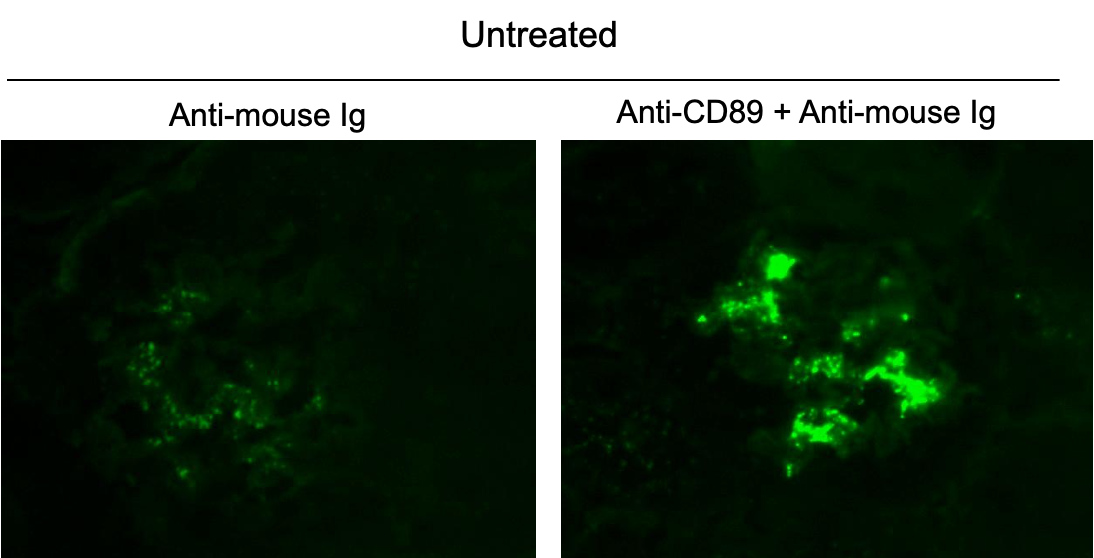


**Supplementary Figure 2**: Immunostaining of kidney cryostat sections from untreated humanized IgAN mice (α1KI-CD89Tg) using anti-CD89 mouse monoclonal antibody (clone A3) plus a secondary anti-mouse antibody coupled to FITC. Negative control was performed with secondary antibody alone.


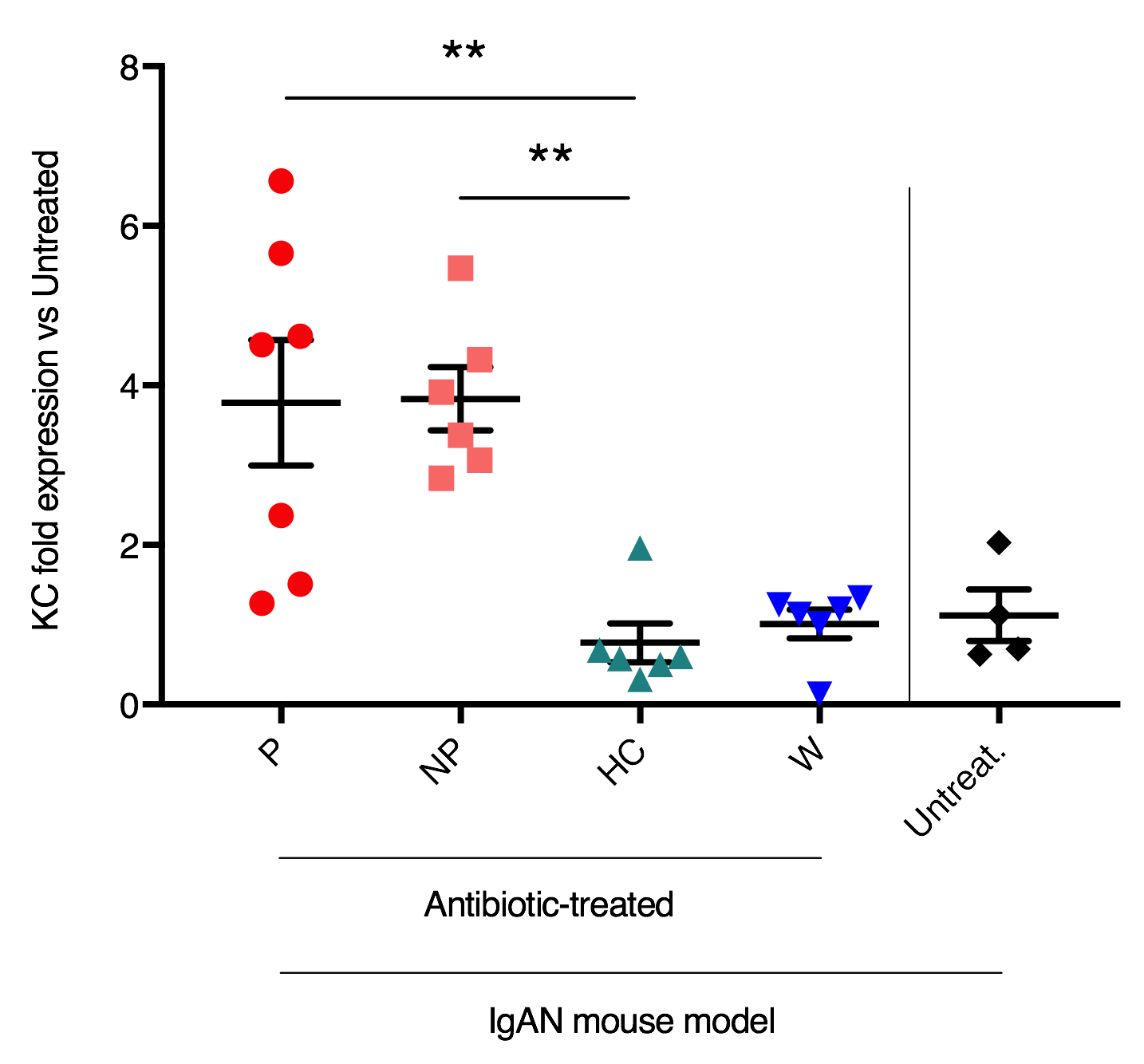


KC fold expression

Antibiotic treated

**Supplementary Figure 3:** Kidney mRNA levels of KC chemokine were evaluated by quantitative RT-PCR. Relative mRNA expressions are displayed as fold change values normalized to untreated mice. Bars and error bars represent the mean ± SEM. **P ≤ 0.01 (Kruskal-Wallis test with Dunn’s multiple comparisons test).

## Supplementary Tables

**Supplementary Table 1.** Clinical features of Healthy control subjects and Progressor and Non-progressor IgAN patients

|  | |  | | IgAN patients | | | | |  |
| --- | --- | --- | --- | --- | --- | --- | --- | --- | --- |
|  | **Healthy Control subjects** (n=10) | | ***Non Progressors***  (n=10) | | | ***Progressors***  (n=10) | | |  |
|  |  | | **Baseline** | | **End of FU^a^** | | **Baseline** | **End of FU** | |
| Age (years) | 41 ± 2.6 | | 42 ± 2.6 | | | 43 ± 2 | | |  |
| Male (%) | 60 | | 70 | | | 60 | | |  |
| Serum Creatinine (mg/dl) | 0.84 ± 0.03 | | 1.00 ± 0.05 | | 0.99 ± 0.04 | | 1.18 ± 0.1 | 1.46 ± 0.16 | |
| Proteinuria (g/day) | 0.04 ± 0.01 | | 0.5 ± 0.09 | | 0.27 ± 0.07 | | 0.83 ± 0.1 | 1.56 ± 0.23 | |
| MDRD GFR^b^ (mL/min/1.73 m^2^) | 99 ± 4 | | 82 ± 4 | | 83 ± 5 | | 68 ± 6 | 54 ± 5 | |

*Data are expressed as mean*± *SEM (standard error of the mean)*

*^a^ FU: follow-up*

*^b^MDRD GFR: Glomerular Filtration Rate estimated by “the Modification of Diet in Renal Disease” equation*

**Supplementary Table 2. Means of number of identified species (OTUs) and Shannon diversity index of mouse gut microbiota after fecal microbiota transplantation.** Means of number of identified species (OTUs) and Shannon diversity index of microbiota from small intestine (SI) or large intestine (LI) of 13-week-old α1^KI^CD89^Tg^-mice fed with water (W) or with fecal microbial cell suspensions from healthy control subjects (HC), progressor or non-progressor IgAN patients (P and NP, respectively). Values within a column with different superscript letters are significantly different (*P* ≤ 0.05; FDR ≤ 0.05).

| **Samples** | **Shannon diversity index** | **OTUs** |
| --- | --- | --- |
| P-SI | 1.77^ab^ | 156.00^ab^ |
| P-LI | 1.96^a^ | 291.00^a^ |
| NP-SI | 1.92^ab^ | 204.67^ab^ |
| NP-LI | 1.36^ab^ | 205.17^ab^ |
| HC-SI | 1.86^ab^ | 193.67^ab^ |
| HC-LI | 1.70^ab^ | 288.83^ab^ |
| W-SI | 1.67 ^ab^ | 166.17^ab^ |
| W-LI | 1.30^b^ | 140.83^b^ |

**Supplementary Table 3. Bacterial genera found in mouse gut microbiota after fecal microbiota transplantation.** Genera found in small intestine (SI) and large intestine (LI) of 13-week-old α1KICD89Tg-mice fed with water (W) or with fecal microbial cell suspensions from healthy control subjects (HC), progressor or non-progressor IgAN patients (P and NP, respectively). Table shows genera statistically significant in at least one condition. (*P means p-value; Student's t-test; bold values denote statistical significance at the P ≤0.05 level).

| Phylum | Family | Genus | P-SI | NP-SI | HC-SI | W-SI | *P** W-SI vs HC-SI | *P*  W-SI vs NP-SI | *P*  W-SI vs P-SI | *P*  HC-SI vs NP-SI | *P*  HC-SI vs P-SI | *P*  NP-SI vs P-SI | P-LI | NP-LI | HC-LI | W-LI | *P*  W-LI vs HC-LI | *P*  W-LI vs NP-LI | *P*  W-LI vs P-LI | *P*  HC-LI vs NP-LI | *P*  HC-LI vs P-LI | *P*  NP-LI vs P-LI |
| --- | --- | --- | --- | --- | --- | --- | --- | --- | --- | --- | --- | --- | --- | --- | --- | --- | --- | --- | --- | --- | --- | --- |
| Actinobacteria | Actinomycetaceae | *Actinomyces* | 0.00 | 0.43 | 0.00 | 0.00 | 0.350 | 0.335 | 0.217 | 0.336 | **0.002** | 0.332 | 0.06 | 0.00 | 0.05 | 0.00 | 0.273 | 0.576 | 0.234 | 0.256 | 0.963 | 0.218 |
|  | Corynebacteriaceae | *Corynebacterium* | 0.00 | 0.73 | 0.01 | 0.00 | 0.370 | 0.340 | 0.373 | 0.347 | 0.447 | 0.341 | 0.15 | 0.00 | 0.08 | 0.02 | 0.270 | **0.032** | 0.198 | 0.188 | 0.539 | 0.161 |
|  | Eggerthellaceae | *Adlercreutzia* | 2.13 | 3.63 | 2.37 | 5.95 | **0.026** | 0.191 | 0.062 | 0.347 | 0.876 | 0.412 | 2.86 | 2.38 | 0.36 | 5.81 | 0.103 | 0.291 | 0.406 | **0.003** | 0.137 | 0.773 |
|  |  | *Eggerthella* | 0.16 | 0.52 | 0.17 | 0.30 | 0.194 | 0.179 | 0.174 | **0.025** | 0.816 | **0.024** | 0.13 | 0.44 | 0.03 | 0.25 | 0.121 | 0.184 | 0.409 | **0.000** | 0.199 | **0.003** |
|  |  | *Slackia* | 1.07 | 1.38 | 0.87 | 2.40 | **0.003** | 0.076 | 0.060 | 0.225 | 0.706 | 0.632 | 0.86 | 0.93 | 0.22 | 1.57 | 0.075 | 0.378 | 0.419 | **0.002** | 0.234 | 0.898 |
| Bacteroidetes | Bacteroidaceae | *Bacteroides* | 0.73 | 2.30 | 2.78 | 0.85 | **0.041** | 0.436 | 0.798 | 0.805 | **0.026** | 0.397 | 1.49 | 0.06 | 1.46 | 0.37 | 0.297 | **0.006** | 0.175 | 0.187 | 0.983 | 0.091 |
|  | Prevotellaceae | *Prevotella* | 0.32 | 0.21 | 0.19 | 0.12 | 0.347 | 0.533 | 0.151 | 0.899 | 0.371 | 0.569 | 0.05 | 0.01 | 0.07 | 0.00 | 0.090 | 0.337 | **0.019** | 0.110 | 0.564 | **0.031** |
| Firmicutes | Erysipelotrichaceae | *Allobaculum* | 39.47 | 25.47 | 25.05 | 37.72 | 0.283 | 0.223 | 0.814 | 0.972 | 0.183 | 0.114 | 15.13 | 58.19 | 23.13 | 57.75 | 0.079 | 0.976 | **0.012** | 0.054 | 0.618 | **0.004** |
|  |  | *Erysipelothrix* | 0.13 | 0.07 | 0.41 | 0.09 | **0.036** | 0.158 | 0.243 | **0.026** | 0.060 | 0.053 | 0.07 | 0.12 | 0.15 | 0.10 | 0.367 | 0.415 | 0.211 | 0.614 | 0.131 | **0.038** |
|  | Eubacteriaceae | *Eubacterium* | 0.10 | 0.04 | 0.44 | 0.09 | 0.060 | 0.068 | 0.909 | **0.035** | 0.062 | 0.067 | 0.09 | 0.06 | 0.16 | 0.11 | 0.377 | **0.030** | 0.561 | 0.105 | 0.270 | 0.487 |
|  | Lachnospiraceae | *Blautia* | 5.32 | 5.29 | 8.32 | 11.05 | 0.387 | **0.022** | **0.041** | 0.307 | 0.351 | 0.988 | 6.80 | 3.83 | 2.39 | 5.64 | 0.177 | 0.470 | 0.742 | 0.336 | 0.150 | 0.340 |
|  |  | *Lachnospira* | 1.06 | 1.49 | 0.41 | 1.72 | 0.184 | 0.823 | 0.568 | 0.078 | 0.393 | 0.627 | 1.81 | 0.71 | 0.24 | 0.61 | 0.267 | 0.801 | 0.274 | 0.191 | 0.153 | 0.319 |
|  |  | *Oribacterium* | 0.30 | 0.44 | 0.35 | 1.23 | 0.078 | 0.113 | 0.061 | 0.701 | 0.833 | 0.544 | 0.43 | 0.23 | 0.03 | 0.25 | 0.081 | 0.900 | 0.504 | **0.023** | 0.112 | 0.438 |
|  |  | *Roseburia* | 0.00 | 0.01 | 0.06 | 0.01 | 0.067 | 0.892 | 0.586 | 0.069 | 0.055 | 0.472 | 0.15 | 0.00 | 0.20 | 0.07 | 0.274 | **0.011** | 0.473 | 0.099 | 0.738 | 0.178 |
|  | Lactobacillaceae | *Lactobacillus* | 21.81 | 18.43 | 12.66 | 9.82 | 0.445 | 0.135 | **0.017** | 0.319 | 0.065 | 0.579 | 30.70 | 18.32 | 37.87 | 12.64 | 0.124 | 0.525 | 0.161 | 0.228 | 0.687 | 0.334 |
|  |  | *Pediococcus* | 0.12 | 0.07 | 0.05 | 0.04 | 0.198 | **0.017** | **0.046** | 0.116 | 0.088 | 0.218 | 1.98 | 0.06 | 0.19 | 0.03 | 0.205 | 0.099 | 0.304 | 0.277 | 0.343 | 0.310 |
|  | Leuconostocaceae | *Leuconostoc* | 0.13 | 0.19 | 0.13 | 0.09 | 0.231 | **0.005** | 0.281 | **0.035** | 0.839 | 0.147 | 0.34 | 0.16 | 0.45 | 0.10 | 0.154 | 0.411 | 0.333 | 0.232 | 0.721 | 0.467 |
|  | Ruminococcaceae | *Faecalibacterium* | 0.16 | 0.27 | 0.63 | 0.07 | 0.350 | 0.344 | 0.459 | 0.564 | 0.442 | 0.653 | 1.83 | 0.01 | 3.23 | 1.13 | 0.339 | **0.005** | 0.581 | 0.152 | 0.572 | 0.160 |
|  | Selenomonadaceae | *Pectinatus* | 0.78 | 0.59 | 0.61 | 0.82 | 0.391 | 0.295 | 0.805 | 0.954 | 0.462 | 0.349 | 0.34 | 1.26 | 0.54 | 1.21 | 0.113 | 0.875 | **0.016** | 0.054 | 0.566 | **0.002** |
| Fusobacteria | Leptotrichiaceae | *Leptotrichia* | 0.00 | 0.28 | 0.00 | 0.00 | 0.341 | 0.341 | 0.341 | 0.348 | 0.908 | 0.347 | 0.03 | 0.00 | 0.04 | 0.00 | 0.341 | 0.341 | 0.341 | 0.341 | 0.867 | 0.341 |
| Proteobacteria | Rickettsieae | *Rickettsia* | 0.01 | 1.47 | 0.00 | 0.01 | **0.042** | 0.341 | 0.951 | 0.339 | 0.070 | 0.341 | 0.01 | 0.00 | 0.02 | 0.01 | 0.195 | 0.166 | 0.631 | **0.044** | 0.123 | 0.431 |
| Tenericutes | Entomoplasmataceae | *Mesoplasma* | 2.39 | 1.32 | 1.36 | 1.96 | 0.307 | 0.204 | 0.342 | 0.952 | 0.087 | 0.039 | 0.78 | 2.47 | 1.07 | 2.54 | 0.094 | 0.898 | **0.015** | 0.072 | 0.700 | **0.005** |
|  | Others | Others | 2.00 | 4.05 | 3.07 | 2.14 | 0.172 | 0.441 | 0.775 | 0.688 | 0.069 | 0.403 | 3.95 | 1.16 | 2.68 | 1.36 | 0.146 | 0.525 | **0.021** | 0.085 | 0.314 | **0.012** |

**Supplementary Table 4 Bacterial species found in mouse gut microbiota after fecal microbiota transplantation.** Bacterial species found in small intestine (SI) and large intestine (LI) of 13-week-old α1KICD89Tg-mice fed with water (W) or with fecal microbial cell suspensions from healthy control subjects (HC), progressor or non-progressor IgAN patients (P and NP, respectively). Table shows species statistically significant in at least one condition. (*P means p-value; Student's t-test; bold values denote statistical significance at the P ≤ 0.05 level).

| Phylum | Family | Species | | P-SI | NP-SI | HC-SI | W-SI | *P** W-SI vs HC-SI | *P*  W-SI vs NP-SI | *P*  W-SI vs P-SI | *P* HC-SI vs NP-SI | *P* HC-SI vs P-SI | *P* NP-SI vs P-SI | P-LI | NP-LI | HC-LI | W-LI | *P*  W-LI vs HC-LI | *P*  W-LI vs NP-LI | *P*  W-LI vs P-LI | *P* HC-LI vs NP-LI | *P* HC-LI vs P-LI | *P* NP-LI vs P-LI |
| --- | --- | --- | --- | --- | --- | --- | --- | --- | --- | --- | --- | --- | --- | --- | --- | --- | --- | --- | --- | --- | --- | --- | --- |
| Bacteroidetes | Bacteroidaceae | | *^a^ B. cellulosilyticus* | 0.02 | 0.00 | 0.07 | 0.01 | 0.390 | 0.542 | 0.467 | 0.359 | 0.526 | 0.339 | 0.60 | 0.00 | 0.19 | 0.08 | 0.376 | **0.000** | 0.322 | 0.150 | 0.448 | 0.260 |
|  |  |  | *B. coprocola* | 0.00 | 0.27 | 0.03 | 0.00 | 0.262 | 0.340 | 0.341 | 0.399 | 0.271 | 0.341 | 0.19 | 0.00 | 0.15 | 0.05 | 0.241 | **0.021** | 0.320 | 0.097 | 0.819 | 0.192 |
|  |  |  | *B. massiliensis* | 0.02 | 0.11 | 0.04 | 0.00 | 0.355 | 0.347 | 0.347 | 0.590 | 0.586 | 0.426 | 0.30 | 0.00 | 0.17 | 0.09 | 0.490 | **0.002** | 0.389 | 0.135 | 0.621 | 0.216 |
|  |  |  | *B. rodentium* | 0.01 | 0.03 | 0.08 | 0.02 | 0.474 | 0.845 | 0.555 | 0.522 | 0.379 | 0.452 | 0.31 | 0.01 | 0.23 | 0.16 | 0.636 | **0.008** | 0.552 | 0.139 | 0.776 | 0.242 |
|  |  |  | *B. uniformis* | 0.02 | 0.02 | 0.13 | 0.00 | 0.333 | 0.341 | 0.307 | 0.411 | 0.419 | 0.942 | 0.74 | 0.00 | 0.51 | 0.23 | 0.438 | **0.001** | 0.422 | 0.165 | 0.743 | 0.251 |
|  |  |  | *B. vulgatus* | 0.00 | 1.55 | 0.08 | 0.00 | 0.154 | 0.340 | 0.610 | 0.365 | 0.150 | 0.340 | 0.26 | 0.00 | 0.24 | 0.06 | 0.381 | **0.004** | 0.427 | 0.263 | 0.949 | 0.320 |
| Firmicutes | Acidaminococcaceae | | *Phascolarctobacterium succinatutens* | 0.01 | 0.01 | 0.02 | 0.01 | 0.564 | 0.758 | 0.770 | 0.696 | 0.680 | 0.978 | 0.31 | 0.00 | 0.08 | 0.08 | 0.910 | **0.034** | 0.398 | 0.264 | 0.428 | 0.268 |
|  | Clostridiaceae | | *Alkaliphilus crotonatoxidans* | 0.15 | 0.34 | 0.08 | 0.19 | 0.264 | 0.543 | 0.737 | 0.243 | 0.385 | 0.420 | 0.05 | 0.26 | 0.09 | 0.11 | 0.707 | 0.199 | 0.235 | 0.107 | 0.071 | **0.045** |
|  |  |  | *Clostridium frigoris* | 0.02 | 0.04 | 0.03 | 0.10 | 0.422 | 0.524 | 0.382 | 0.567 | 0.687 | 0.442 | 4.61 | 0.01 | 0.19 | 0.08 | 0.541 | **0.012** | 0.336 | 0.326 | 0.348 | 0.329 |
|  | Erysipelotrichaceae | | *Allobaculum stercoricanis* | 9.13 | 5.94 | 7.69 | 10.73 | 0.490 | 0.141 | 0.568 | 0.683 | 0.724 | 0.243 | 3.92 | 23.54 | 8.65 | 23.98 | 0.121 | 0.965 | **0.019** | 0.138 | 0.487 | **0.024** |
|  |  |  | *Coprobacillus cateniformis* | 0.07 | 0.11 | 0.72 | 0.07 | **0.043** | 0.474 | 0.906 | 0.055 | **0.043** | 0.395 | 0.02 | 0.13 | 0.04 | 0.04 | 0.902 | 0.404 | 0.372 | 0.383 | 0.431 | 0.291 |
|  |  |  | *Erysipelothrix muris* | 0.25 | 0.11 | 0.69 | 0.21 | **0.022** | **0.029** | 0.555 | **0.008** | **0.036** | **0.037** | 0.19 | 0.35 | 0.35 | 0.35 | 0.999 | 0.995 | 0.231 | 0.999 | 0.494 | 0.144 |
|  | Eubacteriaceae | | *E. biforme* | 0.01 | 0.01 | 0.03 | 0.00 | 0.377 | 0.226 | 0.369 | 0.557 | 0.482 | 0.679 | 0.20 | 0.01 | 0.16 | 0.07 | 0.282 | **0.005** | 0.440 | 0.087 | 0.819 | 0.274 |
|  |  |  | *E. callanderi* | 0.00 | 0.00 | 0.18 | 0.04 | 0.428 | 0.335 | 0.278 | 0.327 | 0.317 | 0.341 | 0.00 | 0.00 | 0.03 | 0.03 | 0.879 | **0.039** | **0.039** | 0.342 | 0.341 | 0.341 |
|  | Lachnospiraceae | | *Bl. coccoides* | 2.29 | 1.66 | 2.40 | 4.39 | **0.034** | **0.015** | 0.075 | 0.307 | 0.902 | 0.529 | 2.34 | 1.99 | 0.77 | 2.31 | 0.052 | 0.723 | 0.973 | 0.068 | 0.078 | 0.720 |
|  |  |  | *Bl. hansenii* | 0.83 | 0.77 | 0.62 | 4.81 | **0.037** | **0.044** | **0.046** | 0.752 | 0.648 | 0.900 | 0.81 | 1.32 | 0.23 | 3.22 | **0.036** | 0.206 | 0.097 | 0.148 | 0.272 | 0.550 |
|  |  |  | *Bl. wexlerae* | 0.04 | 0.07 | 0.21 | 0.10 | 0.414 | 0.529 | 0.210 | 0.295 | 0.219 | 0.419 | 0.80 | 0.05 | 0.41 | 0.33 | 0.721 | **0.038** | 0.287 | 0.079 | 0.394 | 0.094 |
|  |  |  | *Johnsonella ignava* | 0.13 | 0.12 | 0.07 | 2.33 | 0.263 | 0.274 | 0.275 | 0.351 | 0.370 | 0.930 | 0.48 | 0.12 | 0.03 | 0.74 | 0.195 | 0.255 | 0.645 | 0.086 | **0.040** | 0.097 |
|  |  |  | *Lachnospira pectinoschiza* | 1.89 | 2.45 | 0.66 | 4.18 | 0.143 | 0.475 | 0.383 | 0.063 | 0.356 | 0.707 | 3.00 | 1.56 | 0.16 | 1.46 | 0.079 | 0.911 | 0.422 | **0.037** | 0.129 | 0.445 |
|  |  |  | *Oribacterium sinus* | 0.57 | 0.77 | 0.71 | 2.92 | 0.088 | 0.090 | 0.064 | 0.908 | 0.786 | 0.648 | 0.89 | 0.67 | 0.04 | 0.66 | **0.042** | 0.962 | 0.684 | **0.007** | 0.120 | 0.686 |
|  |  |  | *Roseburia faecis* | 0.01 | 0.01 | 0.13 | 0.02 | 0.138 | 0.319 | 0.450 | 0.105 | 0.111 | 0.787 | 0.54 | 0.01 | 0.29 | 0.19 | 0.565 | **0.001** | 0.472 | 0.104 | 0.623 | 0.274 |
|  | Lactobacillaceae | | *Lb. intermedius* | 0.30 | 0.28 | 0.30 | 0.22 | 0.284 | 0.433 | 0.244 | 0.770 | 0.977 | 0.738 | 0.19 | 0.29 | 0.40 | 0.22 | 0.057 | 0.411 | 0.748 | 0.299 | **0.034** | 0.279 |
|  |  |  | *Lb. japonicus* | 0.01 | 0.02 | 0.00 | 0.01 | 0.552 | 0.348 | 0.945 | 0.216 | 0.739 | 0.416 | 2.55 | 0.00 | 0.02 | 0.02 | 0.995 | **0.023** | 0.332 | 0.255 | 0.332 | 0.329 |
|  |  |  | *Lb. salivarius* | 0.02 | 0.37 | 0.01 | 0.01 | 0.900 | 0.115 | 0.208 | 0.116 | 0.351 | 0.124 | 0.06 | 0.08 | 0.40 | 0.00 | 0.227 | **0.009** | 0.305 | 0.333 | 0.307 | 0.716 |
|  |  |  | *Pediococcus argentinicus* | 0.07 | 0.04 | 0.02 | 0.01 | 0.463 | **0.022** | 0.216 | **0.040** | 0.250 | 0.458 | 0.46 | 0.02 | 0.05 | 0.02 | 0.270 | 0.869 | 0.259 | 0.291 | 0.297 | 0.260 |
|  | Leuconostocaceae | | *Weissella salipiscis* | 0.10 | 0.09 | 0.06 | 0.04 | 0.363 | 0.062 | **0.050** | 0.278 | 0.204 | 0.791 | 0.06 | 0.12 | 0.16 | 0.09 | 0.262 | 0.494 | 0.361 | 0.574 | 0.072 | 0.123 |
|  | Ruminococcaceae | | *Anaerotruncus colihominis* | 0.07 | 0.08 | 0.11 | 0.27 | 0.219 | 0.132 | 0.101 | 0.627 | 0.469 | 0.830 | 0.07 | 0.10 | 0.01 | 0.12 | **0.017** | 0.694 | 0.481 | 0.063 | 0.280 | 0.715 |
|  |  |  | *Faecalibacterium prausnitzii* | 0.05 | 0.12 | 0.33 | 0.00 | 0.343 | 0.150 | 0.357 | 0.540 | 0.423 | 0.486 | 1.15 | 0.00 | 0.88 | 0.62 | 0.630 | **0.001** | 0.606 | 0.119 | 0.818 | 0.271 |
|  |  |  | *R. bromii* | 0.03 | 0.01 | 0.06 | 0.00 | 0.341 | 0.288 | 0.236 | 0.470 | 0.678 | 0.535 | 0.26 | 0.00 | 0.35 | 0.19 | 0.448 | **0.000** | 0.734 | 0.112 | 0.762 | 0.228 |
|  |  |  | *R. callidus* | 0.04 | 0.00 | 0.11 | 0.01 | 0.398 | 0.251 | 0.502 | 0.342 | 0.547 | 0.309 | 0.83 | 0.00 | 0.49 | 0.21 | 0.349 | **0.004** | 0.385 | 0.102 | 0.644 | 0.248 |
|  | Selenomonadaceae | | *Pectinatus cerevisiiphilus* | 1.62 | 1.17 | 1.44 | 2.03 | 0.458 | 0.157 | 0.439 | 0.718 | 0.814 | 0.388 | 0.77 | 4.18 | 1.71 | 4.57 | 0.133 | 0.825 | **0.019** | 0.174 | 0.480 | **0.023** |
| Proteobacteria | Comamonadaceae | | *Delftia lacustris* | 0.01 | 0.04 | 0.00 | 0.00 | 0.944 | 0.130 | 0.214 | 0.131 | 0.224 | 0.239 | 0.27 | 0.02 | 0.03 | 0.06 | 0.097 | **0.010** | 0.450 | 0.454 | 0.395 | 0.373 |
|  | Helicobacteraceae | | *Helicobacter mastomyrinus* | 0.06 | 0.27 | 0.10 | 0.17 | 0.149 | 0.504 | 0.095 | 0.251 | 0.506 | 0.191 | 0.09 | 0.04 | 0.00 | 0.01 | **0.022** | 0.453 | 0.084 | 0.294 | 0.051 | 0.391 |
| Tenericutes | Acholeplasmataceae | | *Acholeplasma palmae* | 0.06 | 0.02 | 0.05 | 0.06 | 0.753 | **0.015** | 0.888 | 0.127 | 0.844 | **0.025** | 0.02 | 0.08 | 0.03 | 0.16 | 0.062 | 0.267 | 0.051 | **0.047** | 0.790 | **0.023** |
|  | Entomoplasmataceae | | *Mesoplasma entomophilum* | 4.92 | 2.59 | 3.25 | 4.75 | 0.415 | 0.097 | 0.888 | 0.712 | 0.364 | 0.075 | 1.74 | 8.11 | 3.54 | 9.41 | 0.132 | 0.698 | **0.015** | 0.209 | 0.538 | **0.023** |
| Terrabacteria group | unclassified Terrabacteria group | | *Thermobaculum terrenum* | 0.00 | 0.00 | 0.01 | 0.23 | 0.113 | 0.098 | 0.099 | 0.091 | 0.091 | 0.932 | 0.10 | 0.01 | 0.00 | 0.11 | 0.182 | 0.217 | 0.928 | 0.313 | 0.334 | 0.376 |
|  | Others | | Others | 2.19 | 7.53 | 3.30 | 2.58 | 0.115 | 0.352 | 0.340 | 0.422 | **0.003** | 0.316 | 7.02 | 1.78 | 4.56 | 2.34 | 0.135 | 0.190 | 0.102 | 0.067 | 0.417 | 0.071 |

(a) B., Bacteroides; E., Eubacterium; Bl., Blautia; Lb., Lactobacillus; R., Ruminococcus.

**Supplementary Table 5. Comparison of volatile organic compounds (VOCs) levels (μg/g), in fecal samples of 13-week-old α1KICD89Tg mice fed with water (W mice) or pooled microbial cell suspensions from fecal samples of healthy controls (HC mice), progressor and non-progressor IgAN patients (P and NP mice, respectively).**

|  | P | NP | HC | W |
| --- | --- | --- | --- | --- |
| Alcohols |  |  |  |  |
| 1-Butanol, 3-methyl- | 0.13b | 0.9a | 0.83a | 0.98a |
| 1-Hexanol | 0.3b | 0.63a | 0.58a | 0.3b |
| 1-Octen-3-ol | 0.36b | 1.09a | 0.94ab | 0.68ab |
| 9-Pentadecadien-1-ol | 0.04b | 0.1a | 0.06ab | 0.11a |
| Benzyl alcohol | 0.04bc | 0.12a | 0.06b | nd |
| 1-Hexadecanol | 0.03b | 0.05b | 0.16b | 0.56a |
| Aldehydes |  |  |  |  |
| Butanal, 3-methyl- | 0.15b | 0.61a | 0.15b | 0.27ab |
| Octanal | 0.07b | 0.2a | 0.11ab | 0.12ab |
| 2-Octenal | 0.17b | 0.22b | 0.31a | 0.21b |
| Benzaldehyde | 0.66ab | 1.95a | 0.39b | 0.43b |
| 2-Phenylacetaldehyde | nd | nd | 0.08a | 0.01b |
| 5-Methyl-2-phenyl-2-hexenal | nd | 0.32a | nd | 0.15b |
| Esters |  |  |  |  |
| Propanoic acid, pentyl ester | nd | 0.13a | nd | nd |
| Hydrocarbones |  |  |  |  |
| Tetradecane | 0.17b | 0.57a | 0.25b | 0.67a |
| 8-Heptadecene | 0.03b | 0.08ab | 0.04b | 0.13a |
| Indoles |  |  |  |  |
| Indole | 1.47b | 2.16a | 0.39c | 1.05b |
| 1H-Indole, 3-methyl- | 0.02b | 0.02b | 0.06a | 0.03b |
| Ketones |  |  |  |  |
| 2-Undecanone | 0.09b | 0.26a | 0.32a | 0.24ab |
| 2-Tridecanone | 0.09b | 0.15a | 0.06b | 0.08b |
| 2-Tetradecanone | 0.08ab | 0.08a | 0.03c | 0.04bc |
| Organic acids |  |  |  |  |
| Hexanoic acid | 0.07b | 0.11b | 0.21a | nd |
| Others |  |  |  |  |
| Benzene, 1,3-bis(1,1-dimethylethyl) | 0.65a | 0.35b | 0.44ab | 0.48ab |
| 6,11-Dimethyl-2,6,10-dodecatrien-1-ol | 0.08b | 0.38a | nd | 0.04bc |
| 1H-Indene-4-carboxaldehyde. 2,3-dihydro- | nd | 0.7a | 0.05c | 0.2b |
| Phenols |  |  |  |  |
| Phenol | 0.78a | 0.67a | 0.22b | 0.28b |
| p-Cresol | 0.33a | 0.19bc | 0.25bc | 0.11c |
| Phenol, 4-ethyl- | 0.28a | nd | nd | nd |
| 2-Methoxy-4-vinylphenol | 0.03c | 0.1a | 0.06b | 0.02c |
| Phenol, 2,4-bis(1,1-dimethylethyl) | 0.45a | 0.37ab | 0.29b | 0.24ab |
| Terpenoids |  |  |  |  |
| Farnesene | 0.15a | nd | nd | nd |

nd, not detected.

a–d Values in the same row with different superscript letters differ significantly (P ≤ 0.05) according to one-way ANOVA analysis of variance with a post-hoc Tukey test.
